# Variability of Carbon and Water Fluxes Following Climate Extremes over a Tropical Forest in Southwestern Amazonia

**DOI:** 10.1371/journal.pone.0088130

**Published:** 2014-02-18

**Authors:** Marcelo Zeri, Leonardo D. A. Sá, Antônio O. Manzi, Alessandro C. Araújo, Renata G. Aguiar, Celso von Randow, Gilvan Sampaio, Fernando L. Cardoso, Carlos A. Nobre

**Affiliations:** 1 Centro de Ciência do Sistema Terrestre, Instituto Nacional de Pesquisas Espaciais, Cachoeira Paulista, SP, Brazil; 2 Centro Regional da Amazônia, Instituto Nacional de Pesquisas Espaciais, Belém, PA, Brazil; 3 Instituto Nacional de Pesquisas da Amazônia (INPA), Manaus, Amazonas, Brazil; 4 Embrapa Amazônia Oriental, Belém, Pará, Brazil; 5 Universidade Federal de Rondônia, Porto Velho, Rondônia, Brazil; 6 Universidade Federal de Rondônia, Ji-Paraná, Rondônia, Brazil; 7 Secretaria de Políticas e Programas de Pesquisa e Desenvolvimento, Ministério da Ciência, Tecnologia e Inovação, Brasília, DF, Brazil; Tennessee State University, United States of America

## Abstract

The carbon and water cycles for a southwestern Amazonian forest site were investigated using the longest time series of fluxes of CO_2_ and water vapor ever reported for this site. The period from 2004 to 2010 included two severe droughts (2005 and 2010) and a flooding year (2009). The effects of such climate extremes were detected in annual sums of fluxes as well as in other components of the carbon and water cycles, such as gross primary production and water use efficiency. Gap-filling and flux-partitioning were applied in order to fill gaps due to missing data, and errors analysis made it possible to infer the uncertainty on the carbon balance. Overall, the site was found to have a net carbon uptake of ≈5 t C ha^−1^ year^−1^, but the effects of the drought of 2005 were still noticed in 2006, when the climate disturbance caused the site to become a net source of carbon to the atmosphere. Different regions of the Amazon forest might respond differently to climate extremes due to differences in dry season length, annual precipitation, species compositions, albedo and soil type. Longer time series of fluxes measured over several locations are required to better characterize the effects of climate anomalies on the carbon and water balances for the whole Amazon region. Such valuable datasets can also be used to calibrate biogeochemical models and infer on future scenarios of the Amazon forest carbon balance under the influence of climate change.

## Introduction

The intra-annual variability of carbon and water fluxes over forest and pasture sites in the Amazon region have been reported in many studies in the last several decades. The area covered by the world’s largest tropical forest includes sites with evergreen species, semi-deciduous and transitions to *Cerrado*, among other classifications [1]. Sites with distinct vegetation types or topographies – and subjected to different sums of rainfall – are also different regarding the annual trends of fluxes of carbon, evapotranspiration and sensible heat flux. Southern sites (between latitudes 10° and 20° S) tend to have longer dry seasons while northern locations (between the Equator and 10° S) receive more rainfall due to the proximity to the Intertropical Convergence Zone (ITCZ), a migrating band of clouds and precipitation over the Equator, and the proximity of the Atlantic Ocean where the incoming air provides the moisture that forms precipitation over the Amazon [2]. Different rainfall patterns and annual cycles of air temperature, vapor pressure deficit and incoming solar radiation contribute to different trends in the fluxes of carbon, water and heat between the surface and the atmosphere [3], [4].

Previous works on water and heat fluxes for a group of forest and savanna sites across the Amazon region revealed that evapotranspiration increases during the dry season in sites with higher annual precipitation and shorter dry seasons [5]. This apparent contradiction seems to be explained by the higher availability of incoming radiation and the hypothesized ability of trees for reaching water deep into the soil [1], [6]–[9]. On the other hand, savanna and pasture sites were reported to have decreased evapotranspiration during the dry period [5], [10]. For the carbon fluxes, while an increase in net ecosystem exchange (NEE) during the dry season is reported in some studies [11], [12], others report a decrease of carbon assimilation during the same period [10].

The effects of droughts in 2005 and 2010 on the carbon balance of Amazonian forests have been extensively reported in recent years. A green-up effect following the drought of 2005 [13] was hypothesized to be related to the ability of trees to extract water using deep roots [14]. However, this effect was not observed in another study, which concluded that only 11–12% of Amazonian forests subjected to the drought exhibited greening during the dry season of 2005 [15]. The drought of 2010 was associated with low precipitation in 40% of the vegetated area and low water levels in several rivers of the Amazon basin, such as Rio Negro, near Manaus [16], [17]. As a consequence, a decline of 7% in net primary production (NPP) between July and September of 2010 were reported in a remote sensing study which found that 0.5 Pg C were not sequestered in that year due to the dry conditions [18]. Climate change and droughts in the last decade were related to a decline in global NPP, specifically related to increases in air temperature over the Amazon, which increased autotrophic respiration [19]. Finally, deforestation was also found to play a role in drought events, caused by disturbances in evapotranspiration which affect other regions via regional circulation patterns [20].

In this study, fluxes of carbon dioxide and evapotranspiration were investigated using a dataset composed of seven years of measurements in a forest site within the Jaru Biological Reserve, in Brazil. The time series of fluxes reported here are the longest ever reported for a tropical forest in the southwestern region of the Amazon, enabling the investigation of the impacts on fluxes of extreme climatic events that affected the region, such as the droughts of 2005 and 2010 and the rainy year of 2009 [21]–[25]. The intra-annual variability of carbon flux and evapotranspiration was described using monthly averages and compared to common drivers of the carbon and water cycles, such as air temperature, vapor pressure deficit and incoming solar radiation (direct and diffuse).

The tower is located ≈ 14 km from the site described in other studies of this area [5], [10], [26], making it possible to test the spatial homogeneity of the forest-atmosphere exchanges of carbon and water by the similarity of some results, such as mean daily cycles of fluxes. The partitioning of net ecosystem productivity (NEP) in gross primary production (GPP) and ecosystem respiration (*R_e_*) allowed the investigation of the intra-annual trends of those components of the carbon cycle as well as different metrics of water use efficiency [27]–[29].

## Site and Data

**Figure 1:**
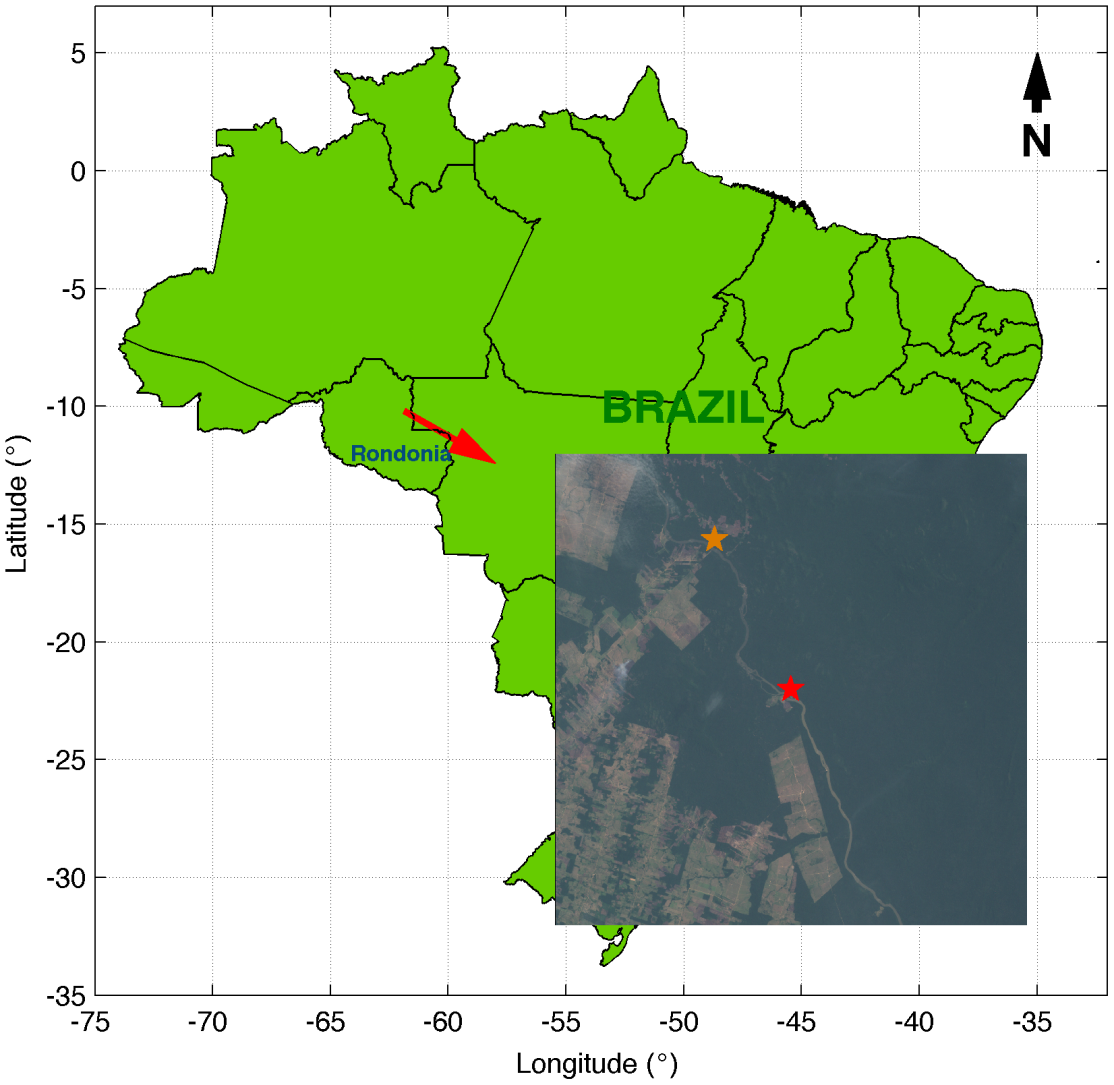
Location of the tower marked with the red star in the detail. The old tower was located at approximately 14 km northwest of the current location (orange star). Satellite picture recorded by Landsat 7 on October 1st 2002.

Measurements were carried out at the Jaru Biological Reserve, near the city of Ji-Paraná, Rondônia, Brazil (Figure 1). Authorization for field studies in this area was provided by IBAMA (National Institute of Environment and Renewable Resources). The tower was mounted in 2004 at the location marked with the red star in Figure 1 (10° 11' 21.2712" S, 61° 52' 15.1674" W, at 145 m above sea level), which is approximately 14 km south-southeast of the old location (orange star, zoomed in detail) up until November of 2002. The old tower, which was disassembled in 2002, was used in several experiments and studies [10], [26], [30]–[39] of the LBA (Large-Scale Biosphere-Atmosphere Experiment in Amazonia) project [30], [34], [40]. The forest was previously characterized as an open tropical rain forest with leaf area index ranging from 4 to 6 m^2^ m^−2^ [30], [41]. Trees are 35 m high, on average, but some reached up to 45 m. Soil depth at the old site ranged from 1 – 2 m and its texture was classified as sandy loam [30].

The new tower was equipped with an eddy covariance (EC) system and micrometeorological sensors. The EC system was installed at 63.4 m and included a 3D sonic anemometer (model Solent 1012R2, Gill Instruments, UK) and an open-path infra-red gas analyzer (IRGA) model LICOR 7500 (LICOR Inc., Nebraska, USA), both operating at 10.4 Hz. A wind vane placed at 62 m (model W200P, Vector Instruments, UK) was used for measurements of wind direction while a barometer (model PTB100A, Vaisala, Helsinki, Finland) was used for recording air pressure at 40 m. Wind speed was measured at several heights above and below the canopy using cup anemometers (model A100R, Vector Instruments, UK) placed at 30, 41, 50.5 and 62.4 m. A vertical profile of thermohygrometers (model Temp107, Campbell, Logan, USA), for measurements of air temperature and relative humidity, was set up at heights 1.6, 11.2, 21.2, 33 and 49 m, while a model HMP45D (Vaisala) was placed at 61.5 m. Incoming and reflected shortwave solar radiation were measured with a pyranometer model CM21, from Kipp&Zonen (The Netherlands), while the incoming and emitted longwave components were measured with a pirgeometer model CG1 (Kipp&Zonen) mounted over arms extending from the tower. Both radiation sensors were installed at 57.5 m. The photosynthetically active component of solar radiation (PAR) was measured with a sensor model SKE 510 (Skye Instruments, UK), also mounted at 57.5 m. Lastly, a vertical profile of intakes for measurements of CO_2_ concentration was setup in 2008 at the following heights: 62, 50, 34, 22, 12 and 2 m.

## Methodology

Fluxes were calculated using the eddy covariance technique [42]–[45], which is implemented in the software Alteddy (Jan Elbers, Alterra Group, Wageningen University, The Netherlands). The software was set up to apply the planar fit rotation [46], [47] to the coordinate system in order to make the vertical velocity zero for different sectors of wind direction. The effects of humidity on the temperature measured by the sonic anemometer were corrected [48] while the influence of air density on the measurements from the infra-red gas analyzer were adjusted using the WPL correction [49]–[51]. In addition, known algorithms were applied to compensate for: a) losses in the high frequency end of the spectra due to spatial separation between sonic anemometer and IRGA [52]–[54]; b) the effects of heating of lenses in the open-path IRGA [55]. Finally, quality control of time series was based on the level of stationarity, i.e., the variability of statistical moments over time [56]–[58]. Stationarity was calculated [59] and summarized in flags from 1 to 9, which are proportional to the level of non-stationarity. For example, fluxes with flags ranging from 1 to 3 may have up to 50% of variability in their statistical moments during the period of 30 min used for averages. Data with flags 1–5 were accepted based on the good energy balance closure of this range, which was previously reported for the old Jaru forest site [26].

Recent developments in the dynamics of air flow past a sonic anemometer’s body led to new findings about the errors of vertical velocity measurements. As a result, different configurations of an anemometer’s transducers (orthogonal or non-orthogonal) may have an impact on fluxes [60], [61]. Those errors can be on the order of ≈10% for sensible heat flux and can propagate to other fluxes through direct measurements or corrections. Due to their recent nature and uncertain impact on fluxes, such corrections were not included in the calculations. However, we expect that the errors calculated in this work (random and gap-filling errors, described next) will account for most of the uncertainty on annual sums of evapotranspiration and carbon fluxes.

The balance of carbon in an ecosystem – the net ecosystem production, NEP – can be calculated as follows:
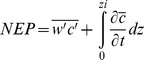
(1)


where *w′* and *c′* are the departures from the mean of vertical velocity and concentration of CO_2_, respectively, *zi* is the measurement height and *z* is the vertical coordinate. Positive values of NEP denote accumulation of carbon by the ecosystem, according to the biological convention. The first term in Equation 1 is the eddy covariance flux, which accounts for the exchanges of carbon by the fast turbulent motions. The second term accounts for the storage of carbon below the measurement point at the top of the tower during conditions of low turbulent motions. The storage of carbon is usually calculated from a vertical profile of CO_2_ concentrations above and below the canopy. The changes in concentration from one half-hour to the next are integrated vertically and contribute to a large fraction of NEP around sunrise due to the stratification of cold – and CO_2_-enriched – air below the canopy during calm nighttime conditions.

The vertical profiles of CO_2_ concentration were not available during the first four years of data, from 2004 to 2007. For this reason, an artificial time series of storage was calculated based on the average values of 2008 to 2010. First, the mean daily cycle of storage was calculated for each month from 2008 to 2010. Next, the daily cycles were grouped by month and averaged over the years. The resulting twelve diurnal cycles were then replicated to fill the respective month, creating a series of 30 minutes averages from January 1^st^ to December 31^st^. The artificial series represented well the real data when comparing the annual impact on the carbon balance: while the average annual sum of storage from 2008 to 2010 was an uptake of ≈1.7 t C ha^−1^ year^−1^, the annual sum resulting from the artificial storage was of ≈1.9 t C ha^−1^ year^−1^. The contribution of the artificial storage is likely to be small to the carbon balance of the first years since its magnitude is close to the average uncertainty derived from the error analysis and gap-filling, which was estimated to be ≈ ±1.7 t C ha^−1^ year^−1^.

**Figure 2:**
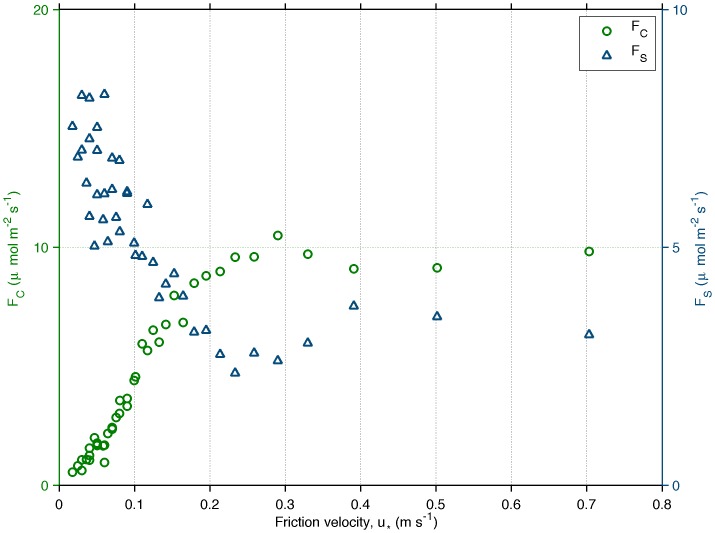
CO_2_-flux (F_C_) and storage (F_S_) plotted versus classes of friction velocity. Atmospheric convention used to denote nocturnal respiration as positive. Only data from 2009 was used for the averages since this was the year with longer data availability of vertical profiles of CO_2_.

The validity of the eddy covariance method relies on the sufficient intensity of wind speed and turbulence in the surface layer, so that vertical exchanges can be averaged over several vortices passing by the tower [62]. The level of turbulence can be inferred by the value of u_*_, the friction velocity, which is calculated as 
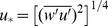
, where 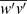 is the longitudinal vertical flux of momentum. The transversal component of the vertical flux 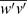 is ignored after rotating the coordinate system to follow the average wind direction [47]. Nighttime conditions usually have light winds and low levels of turbulence, resulting in underestimated fluxes and high values CO_2_ storage below the measuring height (Figure 2). The curves in Figure 2 are used to estimate the u_*_-threshold, used to filter out nighttime or daytime fluxes which are later replaced by modeled values in the gap-filling analysis [63], [64]. However, the choice of the threshold can be subjective and may change the carbon balance depending on the fraction of data replaced by models [26], [65]. Here, we chose the threshold as 0.1 m s^−1^, a value that separates the top 60% of the storage values. A test of sensitivity to this choice was made and the results are presented in the section Results and discussion.

In recent years, two additional terms were proposed to equation 1 to account for horizontal and vertical advection, which are contributions to the flux caused by slopes in the terrain around the tower [66]–[71]. An inclined terrain can lead to horizontal and vertical transports of air that are usually small in flat areas and hence not considered in traditional eddy covariance applications. The effects of advection in Amazonian sites were already investigated over sites with complex topography [72], [73]. An indication of the importance of advection to a site is the curve of CO_2_ storage versus friction velocity, as shown in Figure 2. If the storage is high for low values of u_*_, then cold air is not being flushed out of the ecosystem by horizontal transports and thus advection is not occurring. Since this is the case in Figure 2, it is clear that the effects of advection can be ignored for this site.

Finally, the diffuse component of PAR – caused by scattering of light by clouds, aerosols, smoke or other particles [37], [74], [75] – was calculated using total PAR and the clearness index, an indicator of cloudiness used in solar radiation research. This index is defined as the ratio of global solar radiation at the surface over the radiation received from the Sun at the top of the atmosphere [76]. In addition, the calculations make use of characteristics of the site such as solar elevation angle, latitude and declination of the Sun [76].

### Gap-filling and flux partitioning

Gaps in the time series of fluxes (CO_2_, water vapor) and meteorological variables (air temperature, relative humidity, etc.) need to be filled if annual sums of those variables are calculated. Short gaps – up to 1 hour – are filled by linear interpolation. Longer gaps are filled either by using the average daily cycle for each month, for meteorological variables, or by using other algorithms such as look-up tables, for fluxes. Initially, gaps in air temperature and other meteorological variables are filled since they are required in the gap-filling of fluxes. Next, it is applied an algorithm [26], [64], [77] to fill the fluxes of CO_2_ and evapotranspiration. The algorithm is based on a “look-up table” approach, which tries to fill each gap with an average of good records taken on similar environmental conditions of net radiation, air temperature and vapor pressure deficit. The search starts within ±5 days from the gap and extends up to ±100 days, until it finds at least 5 records to average and fill the gap. In years with fewer long gaps, such as 2005 or 2009, the fraction of gaps due to the low quality flag or low u* was ≈40% before the filling process and 10% afterwards. For years with longer gaps due to instrument malfunction or maintenance, the fraction of missing data could reach up to 70% but the filling algorithm was still able to leave 10–20% of gaps. Those remaining gaps were filled with monthly mean daily cycles, for evapotranspiration, or with modeled fluxes for night and day, for CO_2_ fluxes.

Daytime gaps were filled by light response curves of NEP versus PAR for two classes of air temperature: below and above 25 °C. Nighttime fluxes, or ecosystem respiration *R_e_*, was modeled using an Arrhenius function [78]:
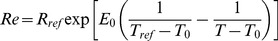
(2)


where *R_ref_* is the respiration at the reference temperature *T_ref_*, which was set to 293.15 K, *E_0_* is the activation energy and *T_0_* is a constant. The constant *T_0_* was set to 227.13 K, as previously reported [78]. The independent variable *T* is referred to air temperature since no measurements of soil temperature were available at the site. The constants *E_0_* and *R_ref_* were calculated by using a non-linear least-squares regression method [64]. The ecosystem respiration calculated in Equation 2 in combination with the gap-filled NEP made it possible to calculate gross primary production (GPP) following the relation NEP  =  GPP – *R_e_*. GPP was used in the analysis of intra-annual variability of fluxes and meteorological drivers, as well as in the calculation of monthly water use efficiency. For the analysis of annual sums (Table 1), the fluxes were integrated from September to August so that one cycle included full wet and dry seasons. This approach will be referred as the seasonal year to distinguish the periods in the discussions that use the regular calendar year. Values computed using the regular calendar year are shown in Table 2.

**Table 1:** Accumulated rainfall and annual sums of total water use (TWU), gross primary production (GPP) and net ecosystem production (NEP) for each seasonal year (integration from September to August).

Year	Rainfall (mm)	TWU (mm)	GPP (t C ha-1 yr-1)	NEP (t C ha-1 yr-1)
2004/2005	1552.8	1095.6±39.8	22.1±0.6	1.7±0.7
2005/2006	1683.8	1000.4±119.1	20.0±1.9	–0.7±1.9
2006/2007	2114.4	1224.7±101.5	22.0±1.1	3.0±1.0
2007/2008	1975	1231.9±88.7	22.0±1.8	4.3±2.6
2008/2009	1964.8	1378.6±61.7	22.7±0.8	6.3±1.3
2009/2010	1861.4	1321.7±41.3	22.7±1.6	4.8±2.5

**Table 2:** Similar to Table 1, but using the calendar year, which uses data integrated from January to December.

Year	Rainfall (mm)	TWU (mm)	GPP (t C ha^−1^ yr^−1^)	NEP (t C ha^−1^ yr^−1^)
**2004**	2181.8	968.8±20.0	29.9±1.2	1.9±1.6
**2005**	1315.2	1172.8±9.1	33.5±0.6	2.2±1.0
**2006**	2075.1	1153.4±48.9	29.7±1.9	–1.2±2.1
**2007**	1942	804.3±22.4	35.7±1.1	4.8±1.1
**2008**	1782.2	1195.0±24.7	36.0±1.7	6.0±2.2
**2009**	2258.4	1498.7±13.4	35.4±0.9	10.4±1.2
**2010**	1551.2	961.7±27.2	38.7±1.8	7.4±2.5

Two metrics of water use efficiency were calculated using NEP, GPP and the amount of water used by the ecosystem in one year, i.e., total water use (TWU). TWU was calculated as the cumulative sum of evapotranspiration, which is also referred as the latent heat flux in atmospheric sciences. The first metric of water use efficiency was GWUE = GPP/TWU and the second was EWUE = NEP/TWU [27]–[29], [79]. While GWUE measures the water use efficiency of the vegetation exclusively, EWUE measures the efficiency of the whole ecosystem, taking into account inputs and outputs of carbon.

The uncertainty in the annual fluxes was estimated by calculating the random (σ_rand_) and gap-filling (σ_gap_) errors. The random error was calculated from the variability of fluxes measured in successive days and under similar conditions [80]. Environmental variables such as net radiation, air temperature and vapor pressure deficit were used to select fluxes subjected to similar physical drivers. The random error σ was estimated as the standard deviation of all differences, normalized by 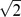 [81]. Then, it was then averaged in several classes and a linear relationship with the magnitude of the CO_2_ flux was found. Next, random noise from a normal distribution with mean 0 and standard deviation σ was created and added to each class of flux, in order to have a synthetic flux with errors. This artificial flux was then gap-filled and its annual sum stored. Those steps were repeated 50 times, generating 50 versions of the original noisy flux, and the uncertainty due to the random error (σ_rand_) was calculated as the standard deviation of all cumulative fluxes. The gap-filling error was calculated by inserting new gaps in the filled flux in the same proportion of the missing data found for day and night after filtering for high quality and turbulent conditions. First, the new random gaps were filled and the annual sum of the flux of CO_2_ was calculated. Then, after 50 iterations, the gap-filling error σ_gap_ was calculated as the standard deviation of all 50 annual sums. The final error for the CO_2_ flux was calculated as 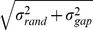.

## Results and Discussion

**Figure 3:**
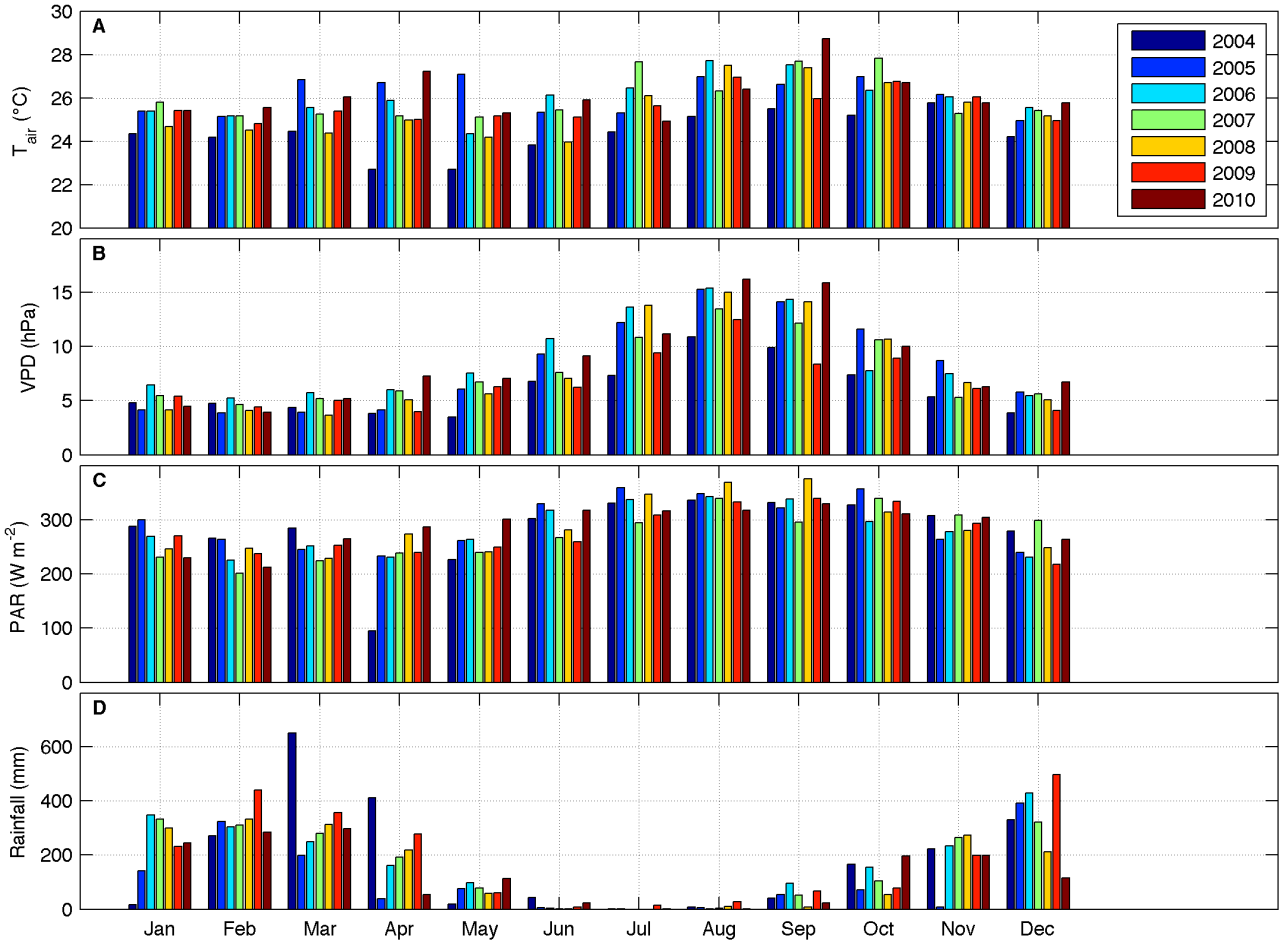
Monthly averages (median) of meteorological drivers. A: air temperature; B: vapor pressure deficit; C: maximum value of incoming PAR at noon; D: accumulated rainfall.

The most important meteorological drivers of fluxes (air temperature, vapor pressure deficit, radiation and precipitation) for the period of 2004 to 2010 are shown in Figure 3. On average, air temperature (Figure 3A) was ≈25°C, from January to March and November to December, and ≈27°C, from July to September. Vapor pressure deficit (Figure 3B) was highest, i.e. drier conditions, from June to October, the same period of the year when incoming PAR was maximal (Figure 3C). The high values of VPD and PAR are in synchronicity with the dry season at this site, as can be noticed by reduced precipitation in the period from May to October (Figure 3D). The remainder of the year is characterized by abundant rainfall, contributing to lower values of air temperature, VPD and PAR, the latter caused by overcast skies.

Abnormal values for some variables are evident in some months when compared to the average of all seven years of data. The period of March to April of 2004 registered two of the three highest values of monthly accumulated precipitation in the dataset. It caused a minimum in PAR for April of 2004 most likely due to cloudy skies. For the following year, 2005, most of the wet season months (Jan, Mar, Apr, Sep, Oct, Nov) had the lowest values of rainfall, in accordance to the extensive drought that affected the Amazon region in this year [23]. Air temperature in March and May of 2005 was the highest among all years. On the other hand, the months of February, March, April and December of 2009 presented high values of accumulated precipitation.

**Figure 4:**
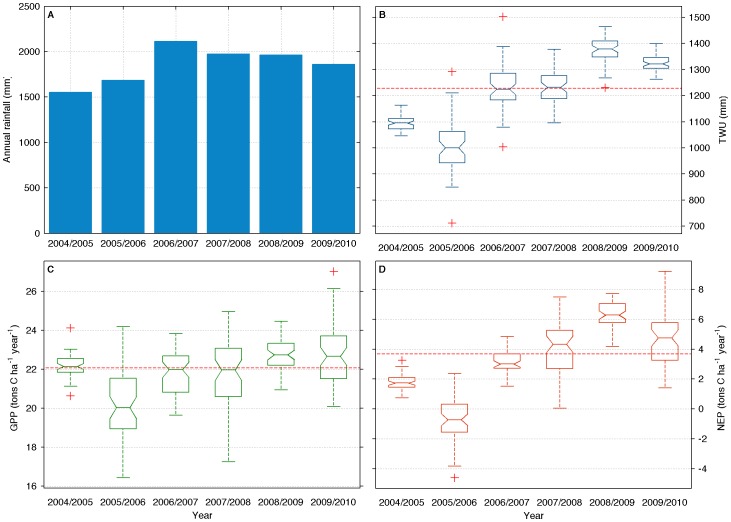
Interannual variability of annual sums for precipitation (A), evapotranspiration, or total water used (B), gross primary production (C), and net ecosystem production (D). Horizontal lines in panels B, C and D denote overall median. Labels in x-axis indicate seasonal year starting in September and ending in August (e.g. from Sep-2004 to Aug-2005).

The cumulative sums of rainfall, TWU, NEP and GPP (Figure 4, Table 1) help to explain the impacts of dry and wet years in the water and carbon cycles. The seasonal year was used in this figure, i.e., integration from September of 2004 to August of 2005. The median value of annual rainfall was 1913 mm, while the minimum was 1552.8 (2004/2005) and the maximum was 2114.4 (2006/2007). The minimum of rainfall was followed by another dry period in 2005/2006, which caused sharp drops in median values of TWU, GPP and NEP. As a result of this long drought, this forest was a net source of carbon for the period of 2005/2006, when NEP was –0.7±1.9 t C ha^−1^ year^−1^. The years that followed this drought had above average precipitation, TWU, GPP and NEP. The peaks in TWU and NEP in 2008/2009 were likely influenced by the high availability of soil water and stand regeneration following three seasonal cycles with high precipitation [82]. The next cycle was characterized by a reduction in the annual rainfall caused by the drought of 2010 in the Amazon [21], which caused drops in TWU and NEP. However, those drops were smaller than the reduction in NEP and TWU immediately after the drought in 2004/2005. It is likely that a further reduction in NEP has occurred in 2011 (data not available), similar to the reduction in 2006.

The annual balances presented in Figure 4 are sensitive to the filtering used to remove nighttime conditions with low levels of turbulence. In general such filtering is based on a threshold of the friction velocity u*, below which values are replaced by modeled data in the gap-filling analysis. The results in Figure 4 are based on a u* threshold of 0.1 m s^−1^. To test the sensitivity of this choice, the threshold was changed to 0.15 m s^−1^ and the resulting carbon balance was calculated. The new threshold changed the annual carbon balance within 41% and 63% of the uncertainty generated by the error and gap-filling analysis, for 2004 and 2005, respectively. Hence, it is unlikely that a higher threshold would change the carbon balance beyond the uncertainty already determined.

**Figure 5:**
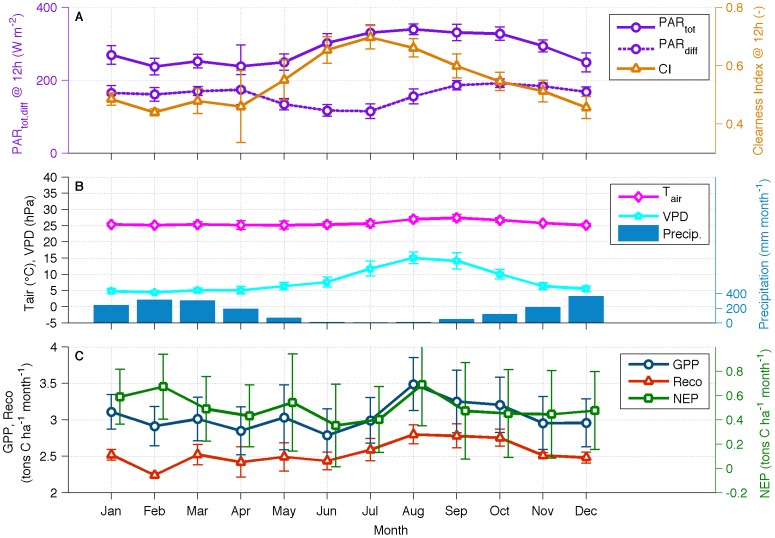
Median intra-annual cycles of PAR (total and diffuse) at noon and clearness index (A); air temperature, vapor pressure deficit and precipitation (B); and gross primary production, net ecosystem production and ecosystem respiration (D).

The average intra-annual variability of some meteorological drivers helped to explain the variability of the carbon and water cycles (Figure 5). Total PAR, represented by the maximum at noon in Figure 5A, is highest in August, near the end of the dry season. The month of August is also the time of the year when the trend of clear-sky conditions reverses, as evident by the decrease in the clearness index and consequent increase of diffuse radiation. Despite the high values of *T_air_* and VPD in August, which forces leaves to close stomata and reduce photosynthesis, GPP and NEP present a temporary maximum at this time of the year. The increased ecosystem production and net uptake of carbon is most likely due to the combination of several factors, such as: the maximum in total PAR; the increase in diffuse PAR – which is known to be highly effective for NEP since light is able to reach leaves not directly exposed to the Sun [74], [75], [83]; new leaves being produced at the end of the dry season; and the availability of water via deep roots. It has been reported before that trees can reach water deep into the soil [7], [14], [84], maintaining high levels of productivity even during the dry season provided that the soil is recharged by rainfall each year during the wet season. Nonetheless, the peak in NEP and GPP was not sustained in September, on average, most likely due to the reduction in the availability of soil water at the end of the dry season.

**Figure 6:**
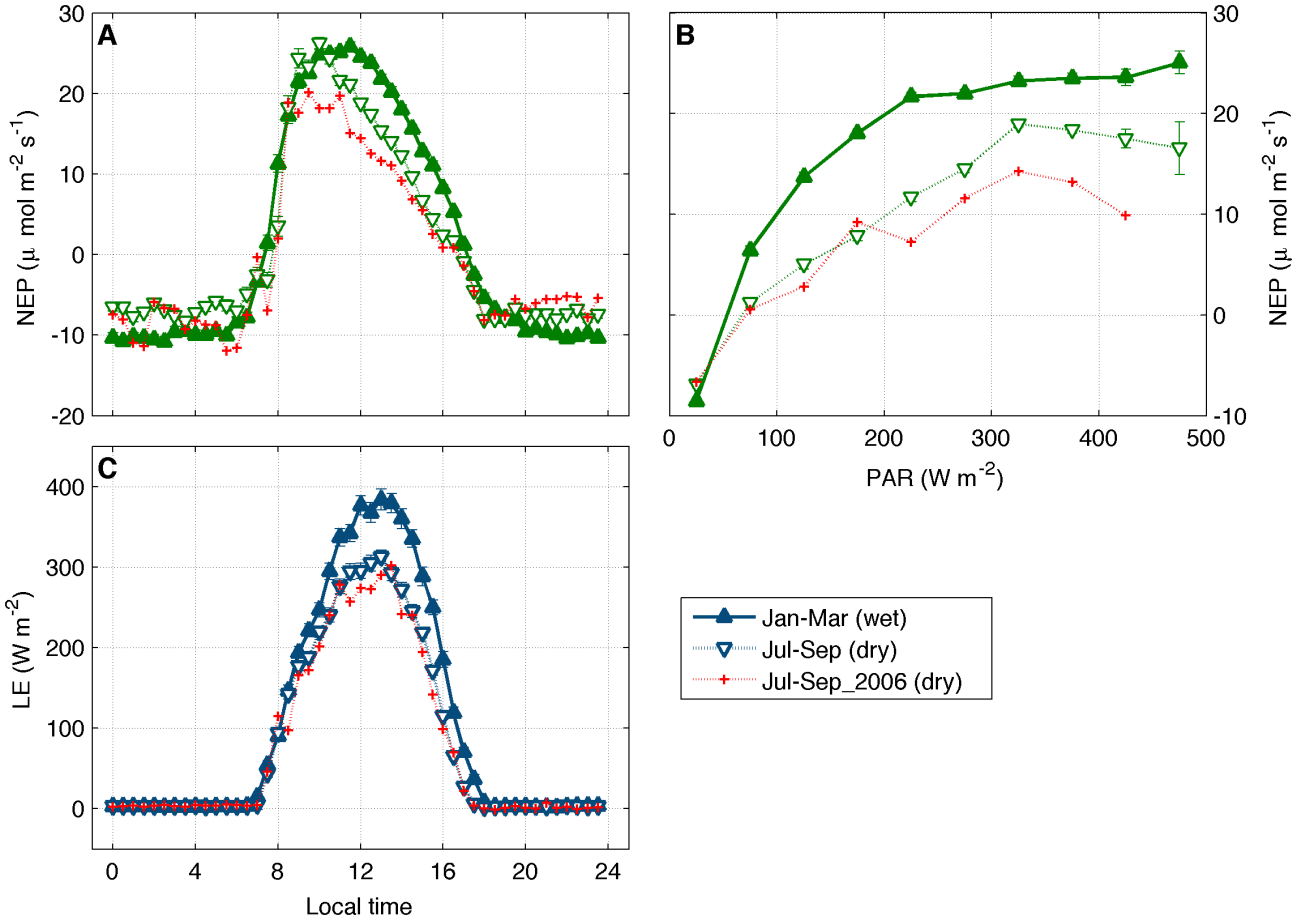
Median daily cycles of net ecosystem production (A), NEP versus incoming PAR (B), and average daily cycles of latent heat flux (C). Measured half-hourly values were averaged for hours of the day (A, C) and for classes of PAR.

Despite the temporary increase in NEP and GPP at the end of the dry season, this ecosystem has lower net uptake of carbon during this period, as can be seen in Figure 6, after averaging the daily cycle of NEP and evapotranspiration over a wet (Jan-Mar) and a dry period (Jul-Sep). Those periods were the same used in a previous accounting of the carbon balance for the old tower at this forest [10]. Daytime NEP is lowest from late morning to late afternoon, indicating lower net uptake of carbon (Figure 6A). The evolution of NEP versus PAR (Figure 6B) confirms the reduction in NEP during the dry season due to a combination of higher temperature, higher VPD and drier soils. In fact, the lower daytime evapotranspiration during the dry season confirms the lower vertical transport of water [85]. Nocturnal values of NEP during the dry season are less negative, indicating lower CO_2_ emissions through soil respiration. This is caused by the drier soil, imposing limitations to microbial activity, which is responsible for CO_2_ emissions from soils. Such lower emissions during nighttime are not enough to offset the lower uptake during daytime, causing the cumulative average NEP during the dry season (1.5 g C m^−2^ day^−1^) to be 21% lower than the cumulative for the wet season (1.9 g C m^−2^ day^−1^).

**Figure 7:**
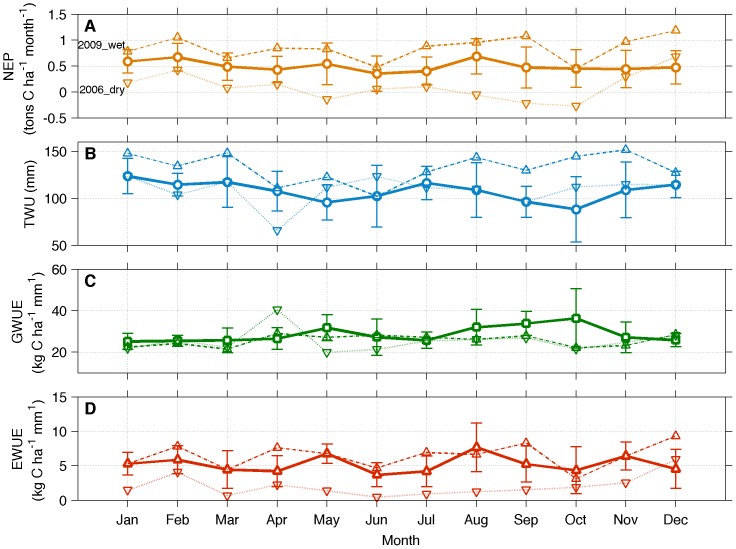
Annual cycles of NEP (A), TWU (B), GWUE (C), and EWUE (D), for average conditions and for years under anomalous climate conditions (dry 2006 and wet 2009). Vertical bars denote the interquartile range.

The dry season of 2006 presented a higher reduction on carbon and water fluxes compared to the usual reduction observed during a typical dry season (Figure 7). The impact of the drought of 2005 was strongest at this ecosystem during the dry season of 2006. Water limitations from September to November of 2005 likely reduced the recharge of soils to the next year. This impact is evident in the average daily cycle of NEP, its relation to PAR, and the daily cycle of latent heat flux (LE) in Figure 6. To further explore the effects of this climate extreme, the monthly variability of NEP and TWU for 2006 was compared with the average cycles and with the wet year of 2009 (Figure 7A, B). In addition, the annual cycles of two water use efficiency ratios were investigated for both extreme years.

Monthly values of NEP were below average for most of 2006, a yearlong effect on fluxes likely caused by the water limitation experienced by this site in 2005 and during the first months of 2006 (February, March and April). This drought reduced net uptake of carbon most likely due to tree mortality and/or decomposition of fallen branches or trees. TWU was below or close to the average for the most part of the first semester of the year. The water use efficiencies (panels 7C,D) were also affected by the dry and wet years. For the dry year of 2006, most of the values of GWUE were below the average due to the decrease of GPP in that year (Figure 4C). For 2009, GPP did not strongly respond to the higher water availability and, when combined with the higher evapotranspiration (Figure 4B), resulted in a GWUE which was also below the average for most of the year. On the other hand, EWUE benefited from the wet year of 2009 with higher values above the monthly means, whereas the drought of 2006 contributed to lower values of EWUE. In conclusion, the drought that affected this forest in 2005 and 2006 strongly influenced the variability of carbon and water fluxes since it altered efficiencies of the ecosystem when using water and accumulating carbon.

## Conclusions

Previous works about the carbon balance of Amazon forest sites revealed that the annual uptake could vary from 1 to ≈6 t C ha^−1^ year^−1^ [10], [86], while climate disturbances, such as droughts, may cause net release of carbon even during the wet season of the year [11]. The determination of the carbon balance of different forest ecosystems in the Amazon requires the long-term studies of fluxes in order to capture the typical conditions as well as transient influences such as droughts or floods. The time series of annual NEP analyzed in this work is the longest ever published for this ecosystem, making it possible to notice the influence of such extreme climate events.

According to results from the first year of measurements at this site [87] – 2004 – evapotranspiration during the dry season (from July to September) decreased by 20%, which is similar to the decrease of ≈ 22% found in this study when comparing the maximum values at midday for wet and dry seasons (Figure 6). In addition, the integration of carbon fluxes in that study resulted in an annual uptake of carbon of ≈5 t C ha^−1^ year^−1^, a value close to the range reported here (4.7±1.7 t C ha^−1^ year^−1^, Table 1).

The carbon flux at this site has a peculiar annual cycle with an increase in net uptake of carbon at the end of the dry season. This lack of synchronicity with the monthly-accumulated rainfall disrupts the positive correlation of the carbon cycle with precipitation. Instead, the effects of incoming solar radiation, new leaves with increased assimilation rates, and probable water use through deep roots [14], [84], enable this ecosystem to increase its net carbon uptake at the end of the dry season. Long-term measurements of soil moisture and soil respiration would surely add valuable information to the carbon and water balances of this ecosystem.

The response of this ecosystem to the climate anomalies of the last decade is in agreement with the results found in many studies of the larger-scale impacts of the droughts of 2005 and 2010 [17]–[20]. The annual carbon balance of this forest (NEP) ranged from a net source, in 2006, to an increasing sink afterwards. A decline in NEP was observed in 2009/2010, probably caused by the drought in 2010. In spite of the typical increase in NEP at the end of the dry season, such mechanism was not enough to revert the long-term rainfall deficit experienced in 2005. In fact, the dry conditions affected the following seasonal year (2005/2006), contributing to turn the ecosystem into a carbon source. Therefore, it is unlikely that a green-up effect could have occurred over this forest [15], [17].

Different parts of the Amazon have distinct annual cycles of meteorological drivers, longer or shorter dry seasons, and consequently different responses to changes in the region’s atmospheric conditions [5], [6], [88], [89]. Based on the variability in the results for NEP, the annual carbon balance is close to 5 t C ha^−1^ year^−1^, with significant changes from year to year depending on climatological conditions. Continuous monitoring of the biogeochemical cycles at this site as well as other ecosystems in the Amazon would help to identify the typical carbon balance, when the ecosystem is not under the influence of climate extremes. Moreover, long-term series of fluxes and meteorological drivers are crucial for the calibration of biogeochemical models that simulate the exchanges of energy and scalars between the biosphere and the atmosphere.
